# Canine Melanoma Immunology and Immunotherapy: Relevance of Translational Research

**DOI:** 10.3389/fvets.2022.803093

**Published:** 2022-02-11

**Authors:** Lidia Tarone, Davide Giacobino, Mariateresa Camerino, Soldano Ferrone, Paolo Buracco, Federica Cavallo, Federica Riccardo

**Affiliations:** ^1^Department of Molecular Biotechnology and Health Sciences, Molecular Biotechnology Center, University of Turin, Turin, Italy; ^2^Department of Veterinary Sciences, University of Turin, Turin, Italy; ^3^Department of Surgery, Massachusetts General Hospital, Harvard Medical School, Boston, MA, United States

**Keywords:** canine melanoma, immunotherapy, vaccination, CSPG4, comparative oncology

## Abstract

In veterinary oncology, canine melanoma is still a fatal disease for which innovative and long-lasting curative treatments are urgently required. Considering the similarities between canine and human melanoma and the clinical revolution that immunotherapy has instigated in the treatment of human melanoma patients, special attention must be paid to advancements in tumor immunology research in the veterinary field. Herein, we aim to discuss the most relevant knowledge on the immune landscape of canine melanoma and the most promising immunotherapeutic approaches under investigation. Particular attention will be dedicated to anti-cancer vaccination, and, especially, to the encouraging clinical results that we have obtained with DNA vaccines directed against chondroitin sulfate proteoglycan 4 (CSPG4), which is an appealing tumor-associated antigen with a key oncogenic role in both canine and human melanoma. In parallel with advances in therapeutic options, progress in the identification of easily accessible biomarkers to improve the diagnosis and the prognosis of melanoma should be sought, with circulating small extracellular vesicles emerging as strategically relevant players. Translational advances in melanoma management, whether achieved in the human or veterinary fields, may drive improvements with mutual clinical benefits for both human and canine patients; this is where the strength of comparative oncology lies.

## Introduction

The interplay between the immune system and cancer has been widely investigated for over a century and has provided the groundwork for the emerging field of research known as immuno-oncology. In this setting, immunotherapy aims to exploit the immune system to change a patient's fate toward cancer eradication and has seen outstanding positive results.

Just like humans, dogs naturally develop a multitude of diseases, including cancer, with six million of new diagnosis each year in the USA alone (1). Developing over a long-time period in an intact immune system, complex interactions between a tumor and the immune system can occur in canine patients, as they do in humans, making cancer cells susceptible to the selective pressure of spontaneous immunity. The similarities between cancer in dogs and humans, and the recent success of immunotherapy in human oncology, have increased enthusiasm for applying it to the treatment of canine cancer. However, advancements in canine medicine are still running behind human clinics, and efforts to develop immunotherapies for dogs are still limited (2). A deeper investigation of the canine immune system and its response to evolving cancers would speed up the development and successful application of immunotherapy in tumor-bearing dogs.

Some of the most relevant comparative aspects of melanoma immunology and immunotherapy will be discussed in this perspective.

Finally, despite several in-human studies have already highlighted the role of small extracellular vesicles (SEVs), including exosomes (EX), in disease progression and their potential as biomarkers in liquid biopsies for diagnosis and therapy, this is a relatively new topic that requires more work before reaching clinical applicability. At the same time, the research in veterinary medicine is still at an early stage, although the potential of SEVs also in this setting is clear (3). As a step forward in this direction, a mention to preliminary examples of the potential of SEVs as promising non-invasive diagnostic and prognostic tools in the melanoma precision veterinary medicine has been added, with potential implications also for the human medicine.

## Canine Oral Malignant Melanoma

Malignant melanoma is among the most common cancers in dogs (4, 5). The most diffuse and fatal subtype is oral malignant melanoma (OMM), which accounts for 30–40% of all canine oral malignancies (6, 7) and 20.3 cases per 100,000 dogs per year (4, 5). OMM is characterized by local invasiveness and high metastatic propensity (6, 8, 9). Up to 74% of OMM rapidly develop distant metastasis, which are the leading cause of death. The survival time of OMM-affected dogs is very short being of about 200 days after diagnosis (8, 10, 11). Surgery is the first-line treatment of choice for the local control of the tumor and correct surgical excision plays a fundamental role in the outcome of the disease (7). It can be flanked by radiotherapy and/or chemotherapy (12). However, metastatic lesions are generally resistant to chemotherapy (4).

Thanks to the release of the canine genome (13), the annotation and deposition of genomic, transcriptomic, and proteomic data derived from canine neoplastic lesions has improved the characterization of the molecular foundations of canine cancers. Although genetic alterations in OMM have not yet been fully described, the mutation profiles of OMM resemble UV-independent molecular etiology, which are typical of human non-UV-induced cutaneous, mucosal, and uveal melanomas (4, 14–16). The same MAPK and PI3K/AKT/mTOR pathways have been found to be activated in OMM and human melanomas, highlighting the overlap between OMM and specific human melanoma subtypes molecular signature. These acquisitions may have an influence on the clinical management of canine OMM, and the first demonstration is the targeted combination treatments with specific human MAPK and PI3K/AKT/mTOR pathway inhibitors that have recently been tested *in-vitro* in the canine setting (17). Of note, one of the most frequently investigated human-melanoma-associated antigens, chondroitin sulfate proteoglycan (CSPG)4, is expressed by most OMM and appears to play a relevant role in clinical outcome, as discussed below (8, 10, 18–20).

Beyond the molecular background, considering the ever more relevant role of immunotherapy in human melanoma management, an increased attention should be dedicated to the immunological aspects of OMM. While the “immune-phenotyping” of human melanoma has been extensively characterized (21), less data are available for OMM. This could slow the development and the translation of effective immunotherapies to veterinary care. Deeper investigations into canine melanoma immunology could guide the application of immunotherapies that have already been approved for use in humans and the possibility of developing novel strategies that could see long-lasting applications in both clinical settings.

## The Immune Landscape of OMM

The OMM immune microenvironment is still widely unexplored. Only recently the interplay between OMM and the immune cells in the tumor microenvironment (TME) has gained attention (22), while several studies have already documented the immunogenicity of human melanoma (23), with a dynamic crosstalk between cells within the TME. Tumor-infiltrating lymphocytes (TILs) are the histopathological reflection of the host's immune response against cancer cells, with the CD3^+^, CD4^+^ and CD8^+^ TILs having a favorable prognostic role in overall survival in the human setting (24).

In a recent study conducted on canine samples, the majority of OMM biopsies were found to be “briskly” infiltrated by different T-lymphocyte subsets (25). A positive correlation between a high TIL level, a high percentage of CD8^+^ T-lymphocytes infiltrating the tumor and better patient survival was observed, which implies the importance of TIL characterization for predicting tumor aggressiveness and prognosis in melanoma-bearing dogs as well (25).

B cells may also play a relevant part in TIL composition. A recent retrospective study conducted on tissue samples collected from canine melanocytic tumors, including the OMM subtype, revealed that there existed a correlation between higher CD20^+^ cell infiltration and the risk of metastasis and tumor relapse (22). Worse survival was observed for dogs whose tumor was more highly infiltrated by CD20^+^ cells, which finally indicates the negative role played by B-lymphocytes in canine melanomas (22). As far as human melanoma is concerned, contradictory roles have been observed for tumor-infiltrating B cells, with both positive and negative correlations with patient clinical outcome being suggested (26–30). Tumor localization and the markers used to detect B cells may be relevant aspects to take into consideration in explaining the discrepancies. A deeper investigation into the role of melanoma-infiltrating B cells in canine and human patients may help in formulating new hypotheses that can be mutually informative in both veterinary and human clinics.

It is also well known that the activation of immunosuppressive cell subpopulations, such as regulatory T cells (Tregs), in human melanoma constitutes an immune-escape mechanism that facilitates tumor growth and progression (31). Starting from this point, a number of studies in veterinary medicine have focused on the characterization of Tregs in canine melanomas (22, 32, 33). An increase in Tregs has been linked to a higher hazard of death in dogs, confirming the connection between Tregs infiltration and worse prognosis (22).

Finally, alterations in peripheral blood leukocytes that mirror systemic inflammation triggered by cancer have already been characterized in human melanoma patients (34). The neutrophil-to-lymphocyte ratio (NLR) and the lymphocyte-to-monocyte ratio (LMR) are prognostic indicators of the evolution of the disease in humans (35, 36), being low LMR counts linked to poorer prognosis in several types of cancers, including metastatic melanoma (37). As such, this aspect is also being investigated in both hematological and solid canine tumors, and LMR is now widely accepted as a prognostic indicator of patients' outcome (38–41). As well, pre-treatment high LMR has been reported to be of significant prognostic value in melanoma-bearing dogs that received anti-PD1 treatment (42), establishing this parameter as a possible indicator of response to immunotherapy. However, neither prognostic nor predictive significance has yet been found for NLR and LMR in a small cohort of canine patients which received anti-CSPG4 immunotherapy (see below), nor any correlations to histological/immunohistochemical parameters of melanoma well-known prognostic factors (43).

Overall, the interest in the contribution of the immune system in shaping the TME in canine patients is growing. The identification of common features in the immunology of human and canine melanoma could help to accelerate the acquisition of novel information that could be exploited to design more effective therapeutic interventions for both settings. Nonetheless, the development of new investigation tools specific for dogs are required to reach the level of knowledge that has been obtained in human oncology.

## Novel Immunotherapeutic Targets For OMM Treatment

Many advancements have been made in immunotherapeutic management of melanoma, especially with the introduction of immune checkpoint inhibitors (ICIs). Regardless of the still-high percentage of patients who do not respond to such therapies (44), ICIs are a true breakthrough in human melanoma treatment, strikingly improving the prognosis of responder patients. Hence, veterinary medicine is now shifting attention to the use of ICIs as a potentially effective systemic treatment also for tumor-bearing dogs.

The expression of Cytotoxic T lymphocyte associated protein 4 (CTLA-4), Programmed death-1 (PD-1) and of PD-1 ligand-1 (PD-L1) on canine immune cells and/or cancer cells has already been investigated and reported (45, 46). Chimeric rat-dog anti-PD-1 and anti-PD-L1 (45) and “caninized” anti-CTLA-4 (46) and anti-PD-1 (42, 47) monoclonal antibodies (mAbs) have been developed. Anti-PD-1 mAbs tested in canine cancer patients, including OMM cases, exerted a significant effect on the inhibition of the PD-1/PD-L1 axis in pilot clinical studies and exhibited remarkable anti-tumor activity, resulting in the increased survival of treated dogs, compared to conventionally treated controls (45, 47).

However, no specific ICI therapy has yet been commercially approved for the treatment of dogs. Nevertheless, in line with human findings (48, 49), veterinary medicine is going toward the characterization of other poorly explored immune checkpoint targets to increase the immunotherapeutic armamentarium. The “next generation immune checkpoints” include B7 homolog 3 protein (B7-H3), lymphocyte activation gene-3 (LAG- 3), T cell immunoglobulin and mucin-domain containing-3 (TIM-3), T cell immunoglobulin and ITIM domain (TIGIT), and CD200. These molecules exert a co-inhibitory function, and strictly act by co-operating with CTLA-4 and PD-1/PD-L1 axes to modulate the anti-cancer immune response (48, 49).

As already demonstrated in humans, TIGIT is upregulated on NK cells of dogs with naturally occurring metastatic osteosarcoma after IL-15 treatment, suggesting a successful possible combinatorial approach for treating metastatic tumors in dogs (50). CD200 blocking in high grade glioma-bearing dogs by means of synthetic peptide ligands, has revealed an increased therapeutic efficacy when combined to an autologous tumor cell lysate vaccine (51). Equally, agonistic mAbs targeting co-stimulatory molecules belonging to the tumor necrosis factor receptor superfamily, such as CD27, OX40, and CD40, have shown impressive anti-tumor effects in pre-clinical and clinical studies (52–54). Interestingly, promising results have been obtained in melanoma-bearing dogs treated with an adenovirus encoding the CD40 ligand (CD40L) (55).

Therefore, targeting different checkpoint molecules and/or finding novel combinatorial strategies in patients which do not respond to PD-1/PD-L1 blockade is essential, and could represent a promising approach to achieve a greater therapeutic effect in a variety of both human and canine tumors.

## B7-H3 Checkpoint Molecule as a Target for OMM

Among the novel immune checkpoints, B7-H3 has recently emerged as an interesting target (56–59). B7-H3 is a type I transmembrane protein member of the B7-superfamily (60). The human B7-H3 gene codes for four immunoglobulin (Ig)-like domains; two pairs of IgV-IgC. The transcribed RNA can be alternatively spliced to generate two proteins, 4IgB7-H3 (B7-H3b) and 2IgB7-H3 (B7-H3) (61). B7-H3 has conserved its amino acid sequence throughout evolution (61, 62) and both the 4Ig- and 2Ig-B7-H3 isoforms are expressed in other species besides humans, including dogs (61, 63). Furthermore, there is 94% amino acid homology between the dog and human B7-H3 sequences (64, 65).

B7-H3 has been suggested to play both a co-stimulatory and inhibitory role in human tumor immunity, depending on the context (62, 66–70). In parallel, the B7-H3 over-expression on tumor cells and its role in promoting tumorigenesis through non-immunologic mechanisms is becoming evident and clinically relevant. The efficacy of the first anti-B7-H3 therapeutic mAb (Enoblituzumab) against B7-H3 expressing tumors, including melanoma, is under examination, alone or in combination with other ICIs, in Phase I-II human clinical trials (71) (https://clinicaltrials.gov/ct2/show/NCT02475213).

**Figure 1:**
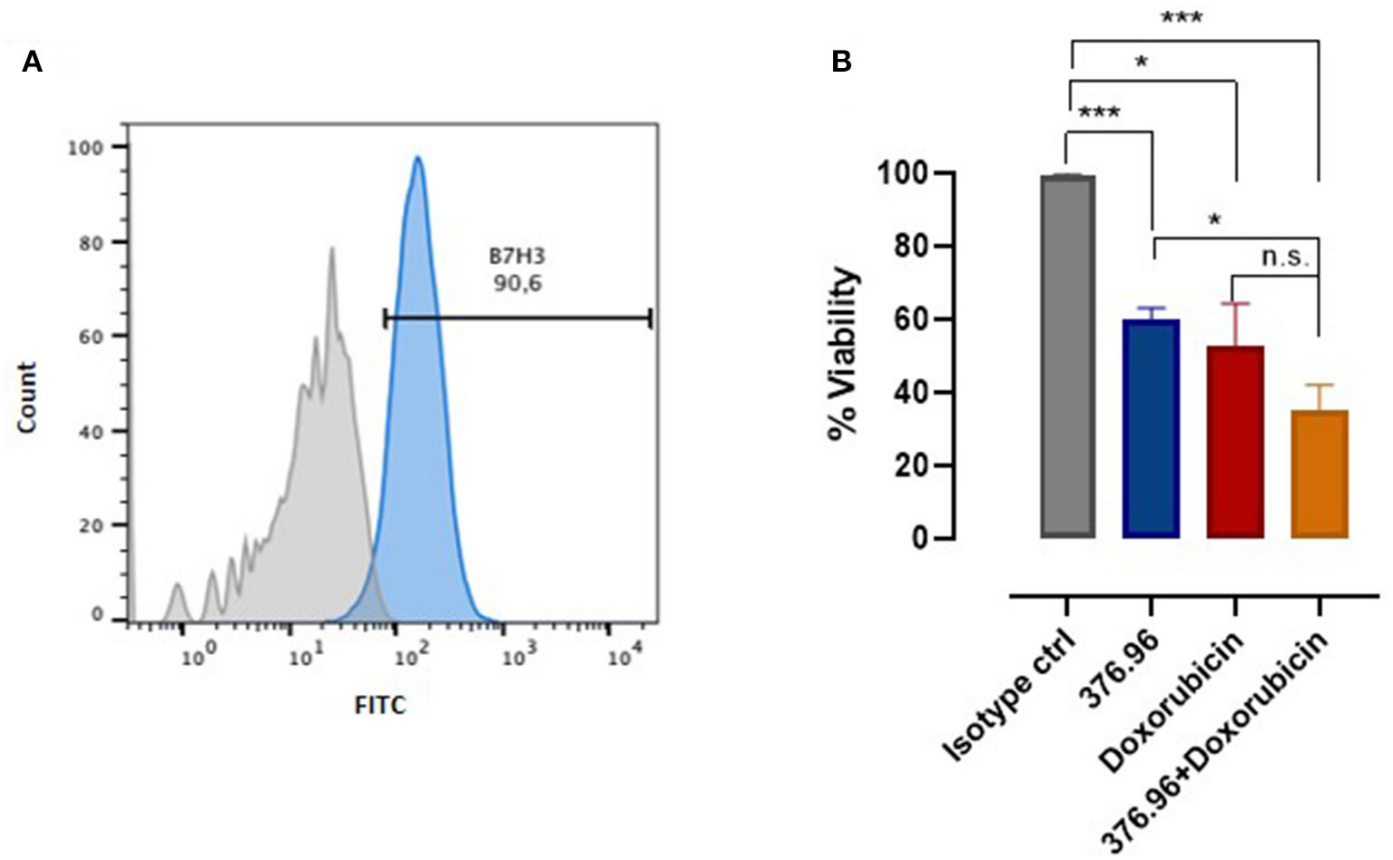
B7-H3 expression and targeting in OMM. **(A)** Flow cytometry analysis of B7-H3 expression on canine CMM-12 OMM cells performed using a FACS Verse (BD Biosciences). The results were analyzed using FlowJo software and representative FACS curves are reported, showing CMM-12 staining using a control IgG1 isotype (filled gray area) and the anti-human B7-H3 mAb 376.96 (filled blue area; 25 μg/ml final concentration). **(B)** Cell proliferation assessed using an MTT assay, as described in (73). The results are expressed as the percentage (mean value ± SEM) of the viability of cells treated with anti-B7-H3 mAb, with respect to cells treated with the control IgG1 isotype, considered as 100%, alone or in combination with doxorubicin at a final concentration of 0.5 μM. Graph shows the results of three independent experiments. Student's *t*-test: *p < 0.01; ***p < 0.001.

B7-H3 role has gained attention also in veterinary oncology. A recent study performed on canine osteosarcoma patients has demonstrated that B7-H3 plays a non-immunological role in sustaining tumorigenicity (72). However, no involvement for B7-H3 in OMM has been reported to date. We found that B7-H3 is expressed by CMM12 (Figure 1A), a canine OMM cell line (9). CMM12 cells that were treated with an anti-human B7-H3 mAb (376.96), which cross-reacted with the canine molecule, displayed a significant reduction in proliferation (Figure 1B), suggesting that B7-H3 downstream signaling potentially sustains OMM cells' proliferative behavior.

Since B7-H3 actively supports tumor cells resistance to chemotherapy (74), we tested the sensitivity of CMM12 to chemotherapy in combination with treatment with the 376.96 mAb. The combinatorial treatment was remarkably more effective than the single treatments alone (Figure 1B). To achieve better and more significant results other combinatorial protocols should be tested, using different mAb and doxorubicin concentrations. Nevertheless, these results prompt further investigations to determine the relevance of blocking B7-H3 to increase the efficacy of chemotherapy for OMM treatment, and we are actively working in this direction. The results obtained in the canine setting could be eventually of clinical relevance for human melanoma treatment too.

## Anti-Cancer Vaccines for OMM Treatment

Therapeutic vaccines against cancer aim to educate the immune system to recognize antigens that are expressed by tumor cells and induce effector immune responses. Several melanoma-associated antigens have been characterized in both humans and dogs, including the disialogangliosides GD2 (75) and GD3 (76–78), tyrosinase (79, 80), gp100 (81), CSPG4 (19, 82), and others.

A number of anti-cancer vaccination strategies targeting these antigens have been tested in companion dogs affected by different tumors, including melanoma (83). These include dendritic cell (DC) vaccines loaded with tumor antigens (84), autologous whole-cell vaccines (81, 85), tumor antigen combined with adjuvants (86) and gene-based vaccination (19, 87); some have already been tested in clinical veterinary trials.

Considering the translational relevance of GD2 and GD3 antigens for both canine and human melanomas, relevant veterinary studies of vaccination against these targets suggested their safety and ability to protect canine melanoma patients when provided as an adjunct to conventional therapies (86, 88). Nevertheless, adjuvants and/or antigen modifications are needed to increase the immunogenicity of the vaccine to be successfully translated in a standard of care (88, 89).

Regarding gene-based vaccination, DNA vaccines against tumor-associated antigens are a promising approach for treating cancer (90, 91). The huge number of DNA-vaccines that have been tested in the last decades in pre-clinical and clinical studies highlights the potential relevance of this strategy for future medical applications. Nevertheless, DNA vaccines face many challenges that till now have prevented their successful translation in the human clinic. Indeed, the first and only FDA-approved DNA vaccine for anti-tumor therapy is ONCEPT (Merial), a xenogeneic DNA plasmid that carries the sequence of human tyrosinase. It was approved for the treatment of dogs affected by locally controlled OMM, since it increased survival times of treated dogs as compared to unvaccinated controls, with no adverse events (92). ONCEPT approval signed the beginning of a new era in treating melanoma in dogs. Nevertheless, some studies have risen controversy around the effectiveness of ONCEPT after its licensing (93, 94).

Whether the human or the veterinary clinical context is concerned, one of the main reasons for the just modest therapeutic effect demonstrated by DNA vaccines in clinical trials could be the strong immunosuppressive condition induced by the tumor. Indeed, a clinically evident tumor triggers several mechanisms of immune suppression that may remain active despite local tumor control. Among them T cell exhaustion and expansion of T regulatory cells, myeloid-derived suppressor cells (MDSCs), and tumor-associated macrophages (TAMs). Several approaches could be applied to enhance vaccine efficacy. Recently, a strong consensus for combining cancer vaccines with ICIs, concomitantly or after immunization (95, 96) is emerging. Various combinatorial strategies have been already tested in pre-clinical and clinical studies for different cancers, resulting promising in coupling the benefits of ICIs in overcoming immunosuppression, with the ability of vaccines to prime the antigen-specific cytotoxic response (97–99).

The combination of immunotherapy with local radiotherapy and/or chemotherapy that can induce immunogenic cell death is likewise a favorable way to prompt a more effective systemic anti-tumor immune response (100–102).

As an example, combinatorial approaches encompassing the use of cytokines, STING agonist and/or vaccines have indeed demonstrated enhanced efficacy when combined to radiotherapy in both pre-clinical and clinical studies, achieving improved therapeutic effects on melanoma-derived metastasis (103, 104). Recently the combination of trimodal radiotherapy and intratumoral immunocytokine vaccination has been tested in advanced stage tumor-bearing dogs, including melanoma cases, and has preliminarily showed to induce positive immunomodulatory effects within the primary tumor (100). All these studies provide the proofs of principle of combining different strategies to achieve better therapeutic effects, eventually allowing to broaden the percentage of patients who might potentially respond to anti-cancer therapy.

In addition to tumor induced immunosuppression, antigen-loss variants of tumor cells may persist after local tumor control that may escape vaccine-induced immune responses. The selection, as vaccination targets, of not disposable tumor associated antigen(s) with a key role in cancer progression may reduce the risk of variant escape. Similarly, the accurate selection of patients for tumor expression of the target antigen would result into more informative clinical trials (105).

## DNA Vaccination Against the CSPG4 Antigen

For its features, we have focused our attention on CSPG4, as a promising tumor-associated antigen to target for effective anti-cancer vaccination against both canine and human melanoma.

CSPG4 is a well-established tumor-associated antigen in human melanomas (106–108), with a widespread expression on cancer cells, but absent in normal adult tissues (108–111). It is highly evolutionarily conserved, with over 88% similarity between the human and canine amino acid sequences (9, 10), suggesting a possible overlapping role in these species. It acts as a scaffold for signaling molecules, forming a complex that drives the activation of key transduction pathways that confer the malignant behavior (108, 112–114). Being a cell-surface tumor antigen, CSPG4 represents an ideal target for effective anti-cancer immunotherapy as CSPG4^+^ cancer cells are potentially susceptible to the concomitant attack of vaccine-inducible T cells and antibodies (20, 91).

As for human melanoma patients, CSPG4 overexpression in canine OMM (18) is clinically relevant, since CSPG4^+^ OMM-affected dogs have worse prognosis than those whose tumors do not express the antigen (9, 19). Therefore, we tested the safety and efficacy of anti-CSPG4 immunotherapy, by means of DNA vaccination, in combination with *in-vivo* electroporation (19, 91, 115) in companion dogs affected by naturally occurring CSPG4^+^ OMM. Dogs affected by a CSPG4-negative OMM were not included in the trials, since they could not benefit from the anti-CSPG4-immunotargeting and would risk leading to confounding results regarding the real vaccination efficacy.

Our first trial with a xenogeneic human (Hu)-CSPG4 DNA plasmid, was safe and effective in inducing a humoral immune response, that was linked to significantly prolonged survival in immunized dogs compared to the conventionally treated population (9, 19, 82). Anti-CSPG4 antibodies induced by the vaccination directly down-regulate CSPG4 expression *in-vitro* hampering CSPG4 tumorigenic functions in melanoma cells, suggesting that they could have a beneficial impact on the clinical course of the disease (9, 19, 82). Immunological mechanisms could be foreseen for vaccine-induced antibodies that could be thus effective in eliminating tumor cells through either antibody-dependent cellular cytotoxicity (ADCC) or complement-dependent cytotoxicity (CDC); this aspect is currently under investigation. Importantly, patients which received the vaccination displayed delayed metastasis onset, compared to non-vaccinated dogs, which rapidly exhibited metastatic spreading (19). Nevertheless, Hu-CSPG4 xenovaccination induced relatively low-affinity antibodies against dog (Do)-CSPG4 (Riccardo et al., manuscript under revision), thus probably limiting the efficacy of the vaccine (116, 117). We have therefore developed a second-generation vaccine that carries a hybrid human/dog sequence, encoding for a chimeric protein that would result in the induction of a more efficient humoral and cellular immune response than those prompted by the fully xenogeneic or fully homologous ones (117, 118). The first demonstration of the potential advantages of applying a chimeric DNA vaccination against the CSPG4 molecule in veterinary medicine is ongoing in a prospective, multi-centric clinical trial in dogs affected by stage II-IV CSPG4^+^ OMM (Riccardo et al., manuscript under revision). To improve the efficacy of this anti-cancer vaccine, the possibility of combining anti-CSPG4 DNA vaccination with ICIs for the treatment of OMM could be an interesting new therapeutic option for canine patients' management, being also of precious translational value for the human context.

## Small Extracellular Vesicles as a New Tool for Melanoma Management

Regardless of the choice of therapy, there is a clear need to identify easily accessible biomarkers that may facilitate the early diagnosis of the disease. In this context, liquid biopsy is emerging as an early, non-invasive, and accessible technique for the accurate molecular profiling of a patient's tumor-derived materials. This technique will likely improve diagnoses, clinical decision making and prognostic accuracy.

Of the tumor-derived materials that can be detected in patient biofluids by means of liquid biopsy, investigations into extracellular vesicles (EVs) are intensifying (119). EVs are lipid-bilayer delimited particles that are naturally shed from cells and are amongst the main key players in cell-cell communication in the TME (120). EVs can carry a heterogeneous variety of biologically active molecules, depending on the cell of origin (121), and play a fundamental role in regulating neoplastic events (122). According to their dimension and biogenesis, we can distinguish EX (ranging from 50 to 150 nm diameter) and microvesicles (ranging from 100 to 1,000 nm diameter), that are emerging as a new frontier in cancer management in humans, with there also being potential impact for dogs (123). The CD9, CD63 and CD81 markers of expression (123, 124) have been usually used as EX biomarkers, although it has to be considered that they are present also on the membrane of other EVs (125, 126), therefore specific markers to strictly discriminate the different subtypes of EVs released by cells are still under discover. For this reason and due to some overlap in size between EX and microvescicles, we will refer more in general to small EVs (SEVs). In oncological patients, SEVs can provide a comprehensive “snapshot” of the tumor status. Knowledge of the proteome of melanoma-derived SEVs is still largely unexplored. However, it has been shown that human-melanoma-cell-derived SEVs-protein cargo differs from normal melanocyte derivatives (120), and potential clinically relevant markers have been identified for isolating circulating SEVs for diagnostic purposes (122). It has been demonstrated that circulating SEVs may provide clinicians with a better overview of dynamic tumor heterogeneity (127), and guide them toward the most appropriate personalized therapeutic approach. Moreover, it has been highlighted the predictive value of circulating SEVs in the melanoma immunotherapy, demonstrating that the monitoring of circulating SEVs-PD-L1 predicts tumor response to treatment and clinical outcome (127).

**Figure 2:**
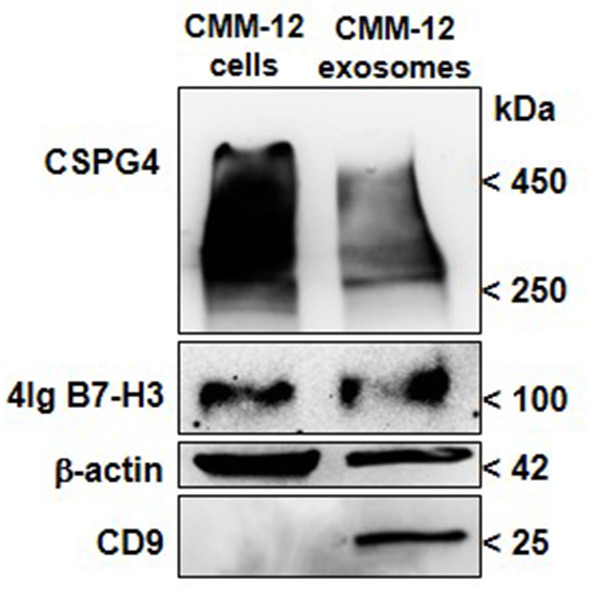
CSPG4 and B7-H3 expression in canine-OMM-cell-derived SEVs. EX purification columns (Exo-spin Midi-Columns, Cell Guidance System, Cambridge, UK) were used to purify enriched EX-SEVs from the supernatant of fetal bovine serum-deprived CMM-12 cells. Briefly, the collected media was centrifuged to remove any cell debris and then incubated with the Exo-Spin Buffer to precipitate SEVs including EX and purified using Exo-Spin midi-column. Eluted SEVs were then ultracentrifuged at 100,000 x g and the pellet was resuspended in RIPA buffer for protein extraction. Representative immunoblot of CSPG4 and B7-H3 of lysates of SEVs is shown. Western Blot analysis for CSPG4 was performed as described in (73), mAb 376.96 was used for B7-H3 detection. CD9 (10626D; Thermo-Fisher Scientific) was used as the SEVs marker and β-Actin (AC-15; Santa Cruz Biotechnology) was used as the protein-loading control.

In canine patients, very few studies have been carried out so far. It has been recently demonstrated that the number of EV isolated from the plasma of dogs with cancer, including melanoma, was higher than in healthy controls (128). On our side, considering that human CSPG4^+^ melanoma cells release SEVs that carry high levels of CSPG4 (111, 129), we investigated its presence in canine-melanoma-cell-derived SEVs. Sustained levels of CSPG4 were found in CMM12-derived SEVs (Figure 2), indicating that circulating-SEV-CSPG4^+^ may be a potential biomarker for canine CSPG4^+^-OMM diagnosis and prognosis for anti-CSPG4 immunotherapy.

Additionally, SEVs that were isolated from human-patient plasma have also been found to be enriched in immunoregulatory proteins (111, 129). Although the immunomodulatory role of B7-H3 in canine cancers still needs to be defined, we sought to discern whether soluble B7-H3 in melanoma-derived SEVs could be detected and potentially used as a prognostic biomarker for OMM. When CMM12-derived SEVs were analyzed, a high level of B7-H3 was detected (Figure 2). This result provides a starting point for investigating the diagnostic and prognostic predictive value of B7-H3 in circulating SEVs in melanoma bearing-dogs, with relevant translational implications for human immunotherapy.

## Discussion

Immunotherapy has revolutionized melanoma treatment in humans and successful clinical responses have been obtained. Spurred by achievements in human clinics, veterinary oncologists have started to exploit the possibility of applying this strategy to the treatment of pet cancers.

While several immunotherapeutic approaches for treating OMM in dogs have been translated from human to veterinary clinics, other strategies have been developed in canine patients first, with high translational relevance for humans (19, 45, 79, 80, 82, 130, 131). Anti-CSPG4 DNA vaccination may emerge as a novel therapeutic approach in veterinary medicine to counteract OMM progression (19, 82), with important implications for human melanoma patients. Nonetheless, the therapeutic effectiveness of our, and other, anti-melanoma strategies could be enhanced. In the wake of human findings, treatments using “old” and “new”-generation ICIs together with anti-cancer vaccination hold great promise for the management of melanoma. In this general framework and considering that precision medicine has become a central theme of cancer management, particular focus must be placed on the key role that SEVs may play in the immuno-oncology of melanoma.

However, several aspects of canine immunity are still unexplored, representing a limitation in the development of effective immunotherapies for dogs. For instance, the lack of a deep characterization of the major histocompatibility complex in dogs, the Dog Leukocyte Antigen (DLA) system, limits the possibilities of developing T-cell-based immunotherapies and investigating functional aspects of the anti-tumor T-cell response *in-vitro*. As well, while the properties of the four human IgG subclasses have been well established and it is known that ADCC and CDC are mainly activated by IgG1 or IgG3, the knowledge about both the complement system in dogs and IgG subclasses is still growing. Up to now, four canine IgG subclasses have been identified, and it is suggested that IgG2 subclasses could mainly provide a specific contribution to ADCC and CDC activity (132). Defining more in detail the components of the canine immune system would allow to better assess the functions of vaccine-induced antibodies for tumor cell killing. In conclusion, exploiting the high similarity between canine and human melanomas, the therapeutic advances achieved in both the veterinary and the human clinics can mutually revolutionize the treatment of melanoma patients.

## Data Availability Statement

The raw data supporting the conclusions of this article will be made available by the authors, without undue reservation.

## Author Contributions

LT, FC, FR, and PB contributed to the conception of this perspective. LT, FC, and FR wrote the draft of the manuscript. DG, MC, SF, and PB contributed to manuscript revision, read, and approved the submitted version. All authors contributed to the article and approved the submitted version.

## Funding

This work was supported by: Italian foundation for cancer research (FR); Fondazione Umberto Veronesi (FR); National Institutes of Health (Grant Number R01DE028172; SF); Fondazione Ricerca Molinette Onlus Torino, Italy (FC); Italian Ministry of Health, within the Progetti ordinari di Ricerca Finalizzata (Grant Number RF-2013-02359216; PB, FC); Faculty resources grant, University of Turin (RILO 2020; FC); Proof of Concept (POC) Instrument Grant, Fondazione Compagnia di San Paolo (FC).

## Conflict of Interest

The authors declare that the research was conducted in the absence of any commercial or financial relationships that could be construed as a potential conflict of interest.

## Publisher's Note

All claims expressed in this article are solely those of the authors and do not necessarily represent those of their affiliated organizations, or those of the publisher, the editors and the reviewers. Any product that may be evaluated in this article, or claim that may be made by its manufacturer, is not guaranteed or endorsed by the publisher.

## Abbreviations

ADCC: Antibody-dependent cellular cytotoxicity
B7-H3: B7 homolog 3 protein
CD40L: CD40-Ligand
CDC: Complement-dependent cytotoxicity
CSPG4: Chondroitin sulfate proteoglycan 4
CTLA-4: Cytotoxic T-Lymphocyte Antigen 4
DLA: Dog Leukocyte Antigen
EVs: Extracellular vesicles
EX: Exosomes
FDA: Food and drug administration
ICIs: Immune checkpoint inhibitors
Ig: Immunoglobulin
LAG3: Lymphocyte-activation gene 3
LMR: Lymphocyte-to-monocyte ratio
mAbs: Monoclonal antibodies
MDSC: Myeloid derived suppressor cells
NLR: Neutrophil-to-lymphocyte ratio
OMM: Oral Malignant Melanoma
PD-1: Programmed death-1
PD-L1: Programmed death-ligand 1
SEVs: Small Extracellular Vesicles
TAM: Tumor-associated macrophages
TIGIT: T cell immunoglobulin and ITIM domain
TIM3: T cell immunoglobulin and mucin-domain containing-3
TIL: Tumor infiltrating lymphocytes
TME: Tumor microenvironment
Tregs: Regulatory T cells.
